# Trypanosomosis: potential driver of selection in African cattle

**DOI:** 10.3389/fgene.2015.00137

**Published:** 2015-04-21

**Authors:** Anamarija Smetko, Albert Soudre, Katja Silbermayr, Simone Müller, Gottfried Brem, Olivier Hanotte, Paul J. Boettcher, Alessandra Stella, Gábor Mészáros, Maria Wurzinger, Ino Curik, Mathias Müller, Jörg Burgstaller, Johann Sölkner

**Affiliations:** ^1^Division of Livestock Sciences, Department of Sustainable Agricultural Systems, BOKU-University of Natural Resources and Life Sciences ViennaVienna, Austria; ^2^Croatian Agricultural AgencyZagreb, Croatia; ^3^Ecole Normale Supérieure, Université de KoudougouKoudougou, Burkina Faso; ^4^Institute of Parasitology, University of Veterinary MedicineVienna, Austria; ^5^Institute of Animal Breeding and Genetics, University of Veterinary MedicineVienna, Austria; ^6^School of Life Sciences, University of NottinghamNottingham, UK; ^7^Animal Production and Health Division, Agriculture and Consumer Protection Department, Food and Agriculture Organization of the United NationsRome, Italy; ^8^FAO/IAEA Joint Division on Nuclear Techniques in Food and AgricultureVienna, Austria; ^9^Parco Tecnologico PadanoLodi, Italy; ^10^Faculty of Agriculture, University of ZagrebZagreb, Croatia; ^11^Biotechnology in Animal Production, Department for Agrobiotechnology, IFA TullnTulln, Austria

**Keywords:** trypanosome, tolerance, Baoule, Zebu, cross, composite

## Abstract

Trypanosomosis is a serious cause of reduction in productivity of cattle in tsetse-fly infested areas. Baoule and other local *Taurine* cattle breeds in Burkina Faso are trypanotolerant. Zebuine cattle, which are also kept there are susceptible to trypanosomosis but bigger in body size. Farmers have continuously been intercrossing Baoule and Zebu animals to increase production and disease tolerance. The aim of this study was to compare levels of zebuine and taurine admixture in genomic regions potentially involved in trypanotolerance with background admixture of composites to identify differences in allelic frequencies of tolerant and non-tolerant animals. The study was conducted on 214 animals (90 Baoule, 90 Zebu, and 34 composites), genotyped with 25 microsatellites across the genome and with 155 SNPs in 23 candidate regions. Degrees of admixture of composites were analyzed for microsatellite and SNP data separately. Average Baoule admixture based on microsatellites across the genomes of the Baoule- Zebu composites was 0.31, which was smaller than the average Baoule admixture in the trypanosomosis candidate regions of 0.37 (*P* = 0.15). Fixation index F*_ST_* measured in the overall genome based on microsatellites or with SNPs from candidate regions indicates strong differentiation between breeds. Nine out of 23 regions had F*_ST_* ≥ 0.20 calculated from haplotypes or individual SNPs. The levels of admixture were significantly different from background admixture, as revealed by microsatellite data, for six out of the nine regions. Five out of the six regions showed an excess of Baoule ancestry. Information about best levels of breed composition would be useful for future breeding ctivities, aiming at trypanotolerant animals with higher productive capacity.

## Introduction

African animal trypanosomosis (AAT) is a severe disease caused by three species of *Trypanosoma* parasites: *T. congolence, T. vivax, and T. brucei*. Trypanosomosis is responsible for the deaths of millions of livestock each year and a reduction in the productivity of many more. With no vaccine available, and with heavy expenditure on trypanocidal and vector control, trypanosomosis is estimated to cost over 4 billion US dollars each year in direct costs and lost production (Hanotte et al., 2003).

West African cattle have the ability to control parasitemia and anemia related to trypanosomosis and a greater ability to grow and produce in tsetse infested areas (Murray et al., 1984). This is thought to result from an adaptation process of indigenous cattle breeds. Trypanotolerant breeds represent a small proportion (6%) of the cattle population of Africa and 17% of the cattle in the tsetse challenged areas (Agyemang, 2005). The option of using those breeds in breeding systems thus reduces or eliminates the use of chemicals to control the trypanosomosis vector and other parasites and contributes positively to a balanced ecosystem health. The complex trypanotolerant trait that is present in indigenous West African taurine cattle is not present in the introgressed *Bos indicus* cattle and is dependent on admixture proportion in hybrid breeds (Freeman et al., 2004).

Humpless taurine populations (*Bos taurus*) are original indigenous cattle of Africa which entered into the African continent before Zebu cattle, around 8000 years ago for longhorn and around 4750–4500 years ago for shorthorn, while the humped zebu populations (*Bos indicus*) were brought only later into the African continent, *via* the Horn of Africa (Loftus et al., 1994; Bradley et al., 1996; MacHugh et al., 1997; Hanotte et al., 2002; Epstein, 1971). It is believed that taurine cattle penetrated West African forests about 4000 years ago (MacDonald and MacDonald, 2002; Freeman et al., 2004) while zebu populations arrived 1300–1000 years ago (Epstein, 1971; MacHugh et al., 1997; MacDonald and MacDonald, 2002).

There is evidence indicating that host genetic factors play a significant role in determining an individual's susceptibility/resistance status to trypanosoma infection (Murray et al., 1984; Hanotte et al., 2003; Courtin et al., 2006, 2007, 2008). Trypanotolerance of cattle can be associated with genomic regioms known as trypanotolerance candidate regions. Observations from Naessens et al. (2002) confirm that this trypanosoma tolerance encompasses at least two mechanisms: one that improves the control of parasitemia and another that limits anemia. The physiological and genetic mechanisms underlying trypanotolerance are being extensively investigated. Hanotte et al. (2003) performed experimental crossing of trypanotolerant N'Dama (*Bos taurus*) and trypanosusceptible improved Kenya Boran (*Bos indicus*) cattle, and mapped QTLs associated to trypanotolerance on 18 autosomes. Results suggest that selection for trypanotolerance within F2 cross between N'Dama and Boran cattle could produce a synthetic breed with higher trypanotolerance levels than currently exist in the parental breeds. Noyes et al. (2010) performed a genetic expression analysis to identify candidate genes in pathways responding to *T. congolense* infection.

Evidence for selective sweeps was observed at TICAM1 and ARHGAP15 loci in African taurine cattle, leading the authors to propose these genes as strong candidates to explain the QTL. Candidate QTL genes were identified in other QTL by their expression profile and the pathways in which they participate. Dayo et al. (2009) tested heterozygosity and variances in microsatellite allelic size among trypanotolerant and trypanosusceptible breeds which led to two significantly less variable microsatellite markers. One of these two outlier loci is located within the confidence interval of a previously described QTL underlying a trypanotolerance-related trait (Hanotte et al., 2003). Stella et al. (2010) analyzed selection signatures by contrasting 32,689 SNP genotypes of trypanotolerant African taurine N'Dama and Sheko cattle with those of all other breeds included in the Bovine HapMap study (Bovine HapMap Consortium, 2009). The overlap of candidate regions found in different studies is comparatively small. West African cattle have the ability to control parasitemia and anemia related to trypanosomosis and a greater ability to grow and produce in tsetse infested areas (Murray et al., 1984). This is thought to result from an adaptation process of indigenous cattle breeds.

Among the indigenous cattle in Burkina Faso, the Baoule, a taurine breed native to the tsetse-challenged southern part of the country, is known for its ability to cope with trypanosome infections. Pure Zebu (Bos indicus) is much more susceptible to the disease, but still preferred by farmers because of body size and suitability as draft animal. With the intention of having both big and tolerant animals, many farmers use composites, continuously mating Zebu, Baoule and their crosses. The preference for larger animals means that Zebu ancestry is predominant among the admixed animals. In genomic regions responsible for trypanotolerance however, higher levels of Baoule ancestry are expected. In a paper on approaches to detect signatures of selection from genome wide scans, Oleksyk et al. (2010) describe a way of detecting significant differences of local admixture levels in crossbred/admixed individuals compared to the average admixture across their genomes. This method can be applied to identify genome signatures of historic selective pressures on genes and gene regions.

Aim of this study was to compare levels of zebuine and taurine admixture in candidate regions for trypanotolerance with the “background” admixture levels, to identify differences in allelic frequencies of trypanotolerant and non-tolerant breeds, and to assess individual differences in admixture for particular animals. Regions potentially responsible for trypanotolerance were identified based on composite log-likelihoods of the differences in allelic frequencies of trypanotolerant and non-tolerant breeds, using Bovine HapMap data (Bovine HapMap Consortium, 2009). Individual admixture levels in these regions versus admixture levels of the background genome of composite Baoule x Zebu animals were compared.

## Materials and methods

### Study design and animals

Blood was taken from 214 animals in total out of which 90 were Baoule from South West (SW) Burkina Faso, 90 were Zebu from the North (*n* = 54) and SW (*n* = 36) regions and 34 were Baoule-Zebu composites from SW. The North of Burkina Faso is part of the Sahelian region with no threat of trypanosomosis, while SW is a Sudanese region that is heavily tse-tse infested.

Designation of animals to breed was based on information by owners of the animals. Animals were from 23 different locations in Burkina Faso. FTA cards were used for collection and storage of blood for all animals.

### Discovery of regions for selective SNP genotyping

Only a small number of SNPs could be selectively genotyped in this project. For the choice of these SNPs, the selection signature approach and sampling of animals by Stella et al. (2010) was employed. Data were from the International Bovine HapMap study (Bovine HapMap Consortium, 2009), including the trypanotolerant African taurine breeds N'Dama and Sheko. Baoule is very closely related to N'Dama (Decker et al., 2014). The 32,689 HapMap SNPs as well as 54,001 Illumina 50k bovine BeadChip SNPs were available for analysis, extending the study of Stella et al. (2010). These two sources of data were merged and after quality control, applying a minor allele frequency threshold of 0.05, a minimum call rate of 0.95 per SNP and removal of duplicate SNP, the final data set comprised 71,235 SNP.

To identify putative selection signatures, allelic frequencies of the N'Dama (*N* = 22) and Sheko (*N* = 19), either pooled or separate, were compared to the allelic frequencies of the entire population (*N* = 497) in the study and nominal *P*-values were calculated for the differences in frequencies at each SNP. The nomimal *P*-values were then used to calculate composite log-likelihoods (CLL) for sliding windows of 9 SNP across the genome. To determine statistical significance, permutation testing was employed by comparing the CLL to the distribution of 50,000 permutations of CLL obtained with random samples of animals (i.e., across all HapMap breeds).

The signals typically pointed to narrow regions (0.2–0.4 Mb), with an average of 254,841 bp. A total of 158 SNPs from 23 regions with strong signals (genome-wide *P* < 0.01 in each breed) were chosen. Within each region, 4–10 roughly equally spaced SNPs were selected from the Illumina data base for genotyping. The rationale for this approach was that signatures of selection are likely linked to trypanosome tolerance in the African taurine breeds. Also, the signals were narrow compared to the results of QTL analyses available at the time, allowing targeted SNP selection. Furthermore, the signatures targeted were observed in both the N'Dama and Sheko, perhaps suggesting that they arose in a past ancestral population of all trypanotolerant breeds and were likely to be present in the Baoule as well.

### Choice of microsatellites

To reflect the admixture levels in the background genome of the animals in this study, a total of 25 autosomal microsatellites were chosen, giving a preference to FAO recommended markers (FAO, 2011), without considering information about trypanosome candidate regions. For the autosomal chromosomes, a total of 31 microsatellite primers have been chosen for the amplification of the genomic DNA. 15 primers were donated by the International Livestock Research Institute, Nairobi, Kenya. PCR conditions were optimized and all the 31 microsatellites tested for polymorphism. A final panel of 25 microsatellites was selected for genotyping of the cattle populations. 22 microsatellites out of them (BM1818, BM1824, BM2113, CSSM066, ETH3, ETH10, ETH185, ETH225, HAUT24, HAUT27, HEL1, HEL5, HEL9, HEL13, ILSTSS005, ILSTS006, INRA023, INRA032, TGLA53, TGLA122, TGLA126, and TGLA227) were from a list recommended by the Food and Agriculture Organisation (FAO) and the International Society for Animal Genetics (ISAG) for use in cattle diversity studies. The others, namely AGLA293, ILSST033, and MGTG4B, were out of both the FAO and the ISAG list. The microsatellites were selected combining information from both National Centre for Biotechnology Information (NCBI, http://www.ncbi.nlm.nih.gov/) database. The selected microsatellites covered 22 autosomal chromosomes.

### Genotyping of animals

Genomic DNA was isolated from white blood cells according to a modified protocol of Whatman (Whatman FTA Protocol BD09). Genotyping of the 25 microsatellites was performed on a MegaBACETM 500 genotyping device. The PCR reaction mixture with the final volume of 22 ɥl included 10 ng template genomic DNA was used in autosomes amplification. 8.05 ɥl of double distilled water, 3.20 ɥl of 10 × Buffer B (Mg2+ free containing 0.8 M Tris-HCl, 0.2 M (NH4)2SO4, 0.2% w/v Tween-20), 2 ɥl of 2 mM dNTP-Mix, 1.60 ɥl of 25 mM MgCl2, 0.5 ɥl of each forward and reverse primers and 0.15 ɥl of 5 U/ɥl FIREoL® DNA polymerase. One primer in each pair was labeled FAM or TET. The 155 selected SNPs were were multiplexed and genotyped on the Sequenom MassARRAY system. The choice of SNPs within the regions of interest was guided by a bioinformatic protocol optimizing the multiplexing strategy. We tried to space the SNPs equally across the 200–400 Kb regions of interest. The total number of SNPs targeted for genotyping was 150 with a minimum of 5 per region. The mastermix for multiplying SNPs comprised 0.50 ɥl of each forward and reverse primers and 0.20 ɥl of 5 U/ɥl HotStar Taq DNA polymerase plus 3 ɥl (3 ɥg) of Salmon sperm to the all mastermix. Total volume of tubes was 4 ɥl. A digestion was made after the PCR with shrimp alkaline phosphate (SAP). The SAP cleaves a phosphate from the unincorporated dNTPs, converting them to dNDPs and rendering them unavailable to future reaction. The SAP mix has been made from 1.5 ɥl of water (HPLC grade), 0.17 ɥl of 10 × SAP buffer, 0.30 ɥl of 1.7 U/ɥl SAP enzyme. 2 ɥl of SAP mix was added to the normal PCR product for digestion. The digestion was followed by ani PLEX PCR. The iPLEX mix was made of 0.619 ɥl of water, 0.20 ɥl of 10 × iPLEX buffer, 0.2 ɥl od iPLEX Termination mix, 0.041 ɥl of iPLEX Enzyme and 0.940 ɥl of the extent primer. 2 ɥl of the iPLEX mix was added to digested product. The following cycling program was run for amplification: 5 min initial denaturation at 95°C followed by 35 cycles of denaturation at 95°C for 1 min, annealing at 55°C for 1:30 min, extension at 65°C for 3 min and final extension step of 65°C for 5 min using Applied Biosystems 96-Well GeneAmp® PCR System 9700 thermal cycler.

The normal PCR of SNP study was run according the following protocol: 2 min initial denaturation at 95°C followed by 45 cycles of denaturation at 95°C for 0:30 min, annealing at 56°C for 0:30 min, extension at 72°C for 1 min and a final extension step of 72°C for 5 min and 4°C for 5 min. The digestion was run on 45 min: 40 min at 37°C and 5 min at 85°C. While the iPLEX PCR run as followed: 030 min of initial denaturation at 94°C, denaturation again at 94°C for 0:05 min, annealing at 52°C for 0:05 min, extension at 80°C for 0:005 min. Annealing up to extension (80°C) was repeated 5 times, from the second denaturation to the extension 40 repeats as well. A final extension step of 72°C was run for 3 min ended by 4°C forever. The normal PCR, the SAP digestion and the iPLEX PCR were performed on using Applied Biosystems 384-Well GeneAmp® PCR System 9700 thermal cycler.

SNPs were positioned according to Btau 4.0. Monomorphic SNPs and SNPs with more than 10% of missing values were excluded, data analysis was performed with the remaining 135 SNPs. Average linkage disequilibrium levels, calculated as R-squared values of SNPs within candidate regions were 0.091, with 5% and 95% quantiles of 0.00003 and 0.478.

### Data analysis

Processing of raw data to formats usable in PLINK was done with SAS (SAS Institute Inc, 2009). Ancestry inferences were performed using STRUCTURE (Pritchard et al., 2000, 2010; Hubisz et al., 2009). STRUCTURE uses a model-based clustering algorithm to infer population structure using genotype data. The software clusters data according to allele frequencies into K populations. As there was linkage disequilibrium in our SNP data, we used version 2.3.4. We employed the admixture model using a burn-in period of 10,000 repeats followed by 10,000 Markov Chain Monte Carlo (MCMC) repeats and considering SNP frequencies correlated. Convergence of the MCMC was investigated with a several STRUCTURE runs on the same datasets. STRUCTURE analyses were all supervised, with added information on pure breed or a cross identity. The assumption of a two breed cross was confirmed with Admixture software (Alexander et al., 2009) with K from 2 to 7, the lowest cross validation error was at *K* = 2; *cv* = 0.48. PLINK (Purcell et al., 2007) was used to recode alleles for analysis. AlphaPhase (Hickey et al., 2011) was used for haplotype imputation of SNPs from candidate regions. To evaluate population differentiation, proc ALLELE of SAS/GENETICS 9.2 was used to calculate the fixation index (F*_ST_*) for every microsatellite, SNP and haplotype derived from candidate regions. This calculation was based on variance in allele frequencies (Weir and Cockerham, 1984; Weir and Hill, 2002).

## Results

Crosses/composites of trypanotolerant and trypanosusceptible cattle were the focus of this analysis. STRUCTURE results indicate that the information acquired from farmers about pure Baoule and Zebu breed types is reasonably accurate with 0.87/0.89 and 0.07/0.06 Baoule ancestry proportions for these two breeds based on Microsatellite/SNP markers (see Table 1). Average Baoule admixture in background genomes (as assessed by microsatellite markers) of Baoule-Zebu composites was 0.31, which was somewhat, but not significantly (*p* = 0.15 based on a *t*-test), smaller than the average Baoule admixture in the AAT candidate regions (assessed by SNP markers), 0.37.

**Table 1:** **Proportions of Baoule admixture calculated with STRUCTURE for Baoule and crosses from Southwest and zebu from Southwest and North**.

**CHR**	**PM**	**M**	***F*ST**	**HF*_ST_***	**Baoule (%)**	**Zebu (%)**	**Comp (%)**
7	5.77–5.98	6	0.18	0.06	0.93	0.39	0.55
7	21.10–21.30	6	0.19	0.15	0.71	0.28	0.48
7	59.30–59.60	7	0.03	0.01	0.49	0.49	0.57
8	55.20–55.60	6	0.16	0.10	0.79	0.21	0.42
16	23.10–23.40	4	0.27	0.26	0.78	0.24	0.46
17	10.60–10.80	6	0.19	0.41	0.89	0.23	0.5
18	12.40–12.60	7	0.13	0.13	0.64	0.34	0.52
18	17.10–17.30	6	0.23	0.18	0.63	0.08	0.23
19	19.70–19.90	6	0.01	0.02	0.47	0.56	0.57
19	27.80–28.00	7	0.03	0.01	0.59	0.40	0.57
20	19.80–20.00	5	0.28	0.17	0.78	0.27	0.35
20	21.09–22.20	4	0.13	0.21	0.68	0.34	0.41
21	11.60–11.80	8	0.17	0.18	0.76	0.22	0.38
21	20.40–20.60	6	0.23	0.18	0.86	0.26	0.54
21	37.90–38.20	7	0.10	0.11	0.53	0.55	0.45
21	42.80–43.00	7	0.12	0.06	0.68	0.26	0.43
21	56.20–56.50	8	0.00	0.01	0.49	0.51	0.52
21	57.40–57.80	7	0.11	0.08	0.73	0.26	0.47
22	20.60–20.90	5	0.20	0.51	0.82	0.17	0.41
22	51.20–51.40	2	0.23	0.33	0.69	0.33	0.55
23	28.80–29.00	4	0.06	0.17	0.51	0.5	0.5
25	15.40–15.70	7	0.11	0.06	0.66	0.27	0.42
26	23.20–23.40	4	0.34	0.15	0.84	0.22	0.41
All SNPs		135	0.14		0.89	0.06	0.37
Microsatellites		25	0.09		0.87	0.07	0.31

*CHR, Chromosome where candidate region is positioned; PM, Position for a corresponding region in Megabases; M, number of markers used in analysis; F*_ST_*, fixation index calculated based on average of SNPs; HF*_ST_*, fixation index calculated for haplotypes; Baoule%, admixture proportion of Baoule in Baoule; Zebu%, admixture proportion of Baoule in Zebu animals; Comp%, admixture proportion of Baoule in composites*.

Admixture proportions were also determined for each genomic region potentially implicated in AAT tolerance (Table 1). Regions with highest Baoule ancestry proportions in the composites were found on chromosome (CHR) 7 [5.77–5.98, 59.30–59.60 Mega bases (Mb)] and on CHR 22 (51.20–51.40 Mb). In some candidate regions, STRUCTURE was not able to cluster individuals, to separate crosses from pure breeds, based on low F*_ST_* values (Table 1). In order to avoid regions with low F*_ST_* values we further considered only the 9 regions where the average F*_ST_* of SNPs by SNP or F*_ST_* of haplotypes (both provided in Table 1) was more than 0.20 (both provided in Table 1). Proportions of individuals with admixture estimates of over 0.60 Baoule ancestry in composites for the nine candidate regions were (in descending order): CHR 22 with 62 and 50% (51.20–51.40 and 20.60–20.90 Mb), CHR 21 (11.60–11.80 Mb) 32%, CHR 26 (23.20–23.40 Mb) 32% CHR 16 (23.10–23.40 Mb) 29%, CHR 20 (19.80–20.00 Mb) 24% and CHR 18 (12.40–17.30 Mb) 18%. Average number of haplotypes per region was 12.59 for Baoule, 11.91 for Zebu and 10.64 for composites. Numbers of haplotypes per breed and genomic region are provided in Table 2. Haplotypes with more than 10% frequency in at least one of the breeds are given in Table 3 for the nine candidate regions with F*_ST_* ≥ 0.20. Reconstructed haplotypes showed differentiation of the Zebu and Baoule individuals. For the region on CHR 21 (20.40–20.60 Mb), the total frequency of two haplotypes (out of 22) was 83.34% for Baoule, in composites the frequency of these haplotypes was 51.52% (they had 13 haplotypes in total) whereas their frequency (16 haplotypes) in Zebu animals was 22.03% (Table 2). Regions on chromosomes 22 (51.20–51.40 and 20.60–20.90 Mb) and 16 (23.10–23.40 Mb) had 45.59% (out of 98.53%), 32.35% (out of 98.53%) and 34.33% (out of 89.55%) of haplotypes predominant in Baoule. Pair-wise chi-squared tests indicated significant differences in haplotype composition for all pairs of breeds (Baoule–Zebu, Baoule–composites, Zebu–composites), in all regions, except for regions on chromosome 20 (19.80–20.00 and 21.90–22.20 Mb), where haplotypes of composites were not significantly different from Zebu (*P* = 0.0896, *P* = 0.1539). Differences were insignificant after applying Bonferroni correction in the region of Chr 18 (12.40–12.60 Mb) for Zebu and composites and region from CHR 22 (51.20–51.40 Mb) of Baoule and composites. F*_ST_* calculated for haplotypes was greatest for CHR 22 (20.60–20.90 Mb), with a value of 0.51.

**Table 2:** **Number of reconstructed haplotypes from SNPs in candidate regions**.

**CHR**	**PM**	**N**	**B**	**Z**	**Comp**
7	5.77-5.98	2	4	4	4
7	21.10-21.30	3	7	5	6
7	59.30-59.60	4	14	15	12
8	55.20-55.60	6	22	17	18
16	23.10-23.40	3	8	7	7
17	10.60-10.80	5	8	6	6
18	12.40-12.60	4	12	11	11
18	17.10-17.30	5	15	15	11
19	19.70-19.90	6	12	11	9
19	27.80-28.00	7	15	21	12
20	19.80-20.00	4	10	9	10
20	21.09-22.20	4	6	8	6
21	11.60-11.80	6	22	16	13
21	20.40-20.60	6	7	10	10
21	37.90-38.20	5	10	17	12
21	42.80-43.00	5	13	13	14
21	56.20-56.50	7	20	20	15
21	57.40-57.80	7	29	19	19
22	20.60-20.90	4	4	4	3
22	51.20-51.40	2	2	3	3
25	28.80-29.00	6	25	19	21
26	15.40-15.70	4	12	12	12
Total		105	277	262	234

*CHR, Chromosome where candidate region is positioned; PM, Position for a corresponding region in Megabases; N, number of SNPs in candidate region used to reconstruct haplotype; B, number of haplotypes found in pure Baoule animals; Z, number of haplotypes found in pure Zebu animals; Comp, number of haplotypes found in composites*.

**Table 3:** **Most frequent haplotypes**.

	**Baoule**			**Zebu**		**Composite**	
**CHR**	**H**	**N**	**% f**	**N**	**% f**	**N**	**% f**
**16 (23.10–23.40 Mb)**							
	000	68	37.78	1	0.57	10	14.93
	001	16	8.89	2	1.14	3	4.48
	011	59	32.78	11	6.25	13	19.40
	110	15	8.33	100	56.82	25	37.31
	111	6	3.33	41	23.30	12	17.91
	00110	7	3.89	108	60.00	19	27.94
**17 (10.60–10.80 Mb)**							
	11110	150	83.33	59	32.78	38	55.88
	01001	10	6.45	28	16.00	8	12.70
**18 (17.10-17.30 Mb)**							
	01101	7	4.52	80	45.71	18	28.57
	11001	52	33.55	15	8.57	13	20.63
	11101	6	3.87	9	5.14	7	11.11
	11111	52	33.55	3	1.71	5	7.94
	0001	10	5.92	13	7.34	2	3.13
**20 (19.80–20.00 Mb)**							
	0011	93	55.03	19	10.73	13	20.31
	0111	13	7.69	1	0.56	1	1.56
	1001	3	1.78	32	18.08	12	18.75
	1011	24	14.20	27	15.25	3	4.69
	1101	5	2.96	54	30.51	18	28.13
	1111	6	3.55	22	12.43	10	15.63
	0111	27	15.00	2	1.11	1	1.47
**20 (21.09–22.20 Mb)**							
	1011	10	5.56	74	41.11	20	29.41
	1111	137	76.11	72	40.00	38	55.88
	010011	6	3.41	52	29.38	14	21.21
**21 (20.40–20.60 Mb)**							
	011111	75	42.61	9	5.08	11	16.67
	110011	8	4.55	71	40.11	7	10.61
	111111	81	46.02	30	16.95	23	34.85
	0101	21	11.67	1	0.56	1	1.47
**22 (20.60–20.90 Mb)**							
	1101	118	65.56	17	9.44	22	32.35
	1111	40	22.22	159	88.33	44	64.71
	01	61	33.89	136	75.56	36	52.94
**22 (51.20–51.40 Mb)**							
	11	119	66.11	30	16.67	31	45.59
	0000	10	5.92	60	40.00	18	27.69
**26 (23.80–29.00 Mb)**							
	0001	3	1.78	17	11.33	6	9.23
	0010	2	1.18	25	16.67	6	9.23
	1101	64	37.87	4	2.67	12	18.46
	1111	42	24.85	13	8.67	8	12.31

*CHR, Chromosome and region in Mb of candidate regions H- Reconstructed haplotype; N, Number of haplotypes; %f, frequency of the haplotypes; In table are shown haplotypes with >10% frequency in at least one of the studied populations*.

Genes found in candidate genomic regions studied as recovered from Ensembl (www.ensembl.org) are provided in Supplementary Table 1. Their potential relevance to trypanotolerance based on information from other studies is discussed below.

## Discussion

Baoule admixture across the genome, based on a sample of 25 microsatellite markers, of the Baoule-Zebu composites was 0.31, compared to the average Baoule admixture based on SNPs in AAT candidate regions (0.37), see Table 1. This difference was not significant, though (*P* = 0.15). Admixture was measured with two distinct types of markers, justification for the process can be found in a study of Schopen et al. (2008), showing that the information content of one microsatellite corresponds to an equivalent to that of about three SNPs in cattle. Similar results were provided by Gärke et al. (2012) when analyzing population differentiation of chicken breeds. The average number of haplotypes per region was 12.59 for Baoule, 11.91 for Zebu and 10.64 for composites. We found slightly more haplotypes in our candidate regions for Baoule (277) compared to Zebu (262), the lower number of haplotypes in composites is most likely due to the smaller sample size. The results are in contrast to Murray et al. (1984) who found higher diversity in *B. indicus* compared to *B. taurus*. It is known that the West African *B. taurus* populations contain a degree of *B. indicus* admixture (Alvarez et al., 2014), but the proportion is small in Baoule (Hanotte et al., 2003; Soudré et al., 2013).

Due to the very low F*_ST_* in some candidate regions, STRUCTURE was not able to separate pure breeds in those regions. Overall F*_ST_* calculated with SNPs from candidate regions (F*_ST_* = 0.14) matches that from other studies, see Dayo et al. (2009), while F*_ST_* calculated with microsatellites (F*_ST_* = 0.09) was lower. When looking at F*_ST_* for single SNPs, the highest value was 0.70 for a SNP on CHR 26 while very low F*_ST_* values were found in almost every region (results not shown). F*_ST_* values below 0.05 indicate low differentiation, 0.20 moderate to strong and values above 0.65 indicate extreme differentiation (Barreiro et al., 2008). Therefore we concentrated on candidate regions which showed F*_ST_* > 0.20. Using information from sex linked markers Soudre (2011) found a relative age of admixture of 69 ± 43 years from 2007 data for the crosses analyzed in this study. This is consistent with the findings of Grace (2006) who described the history of introduction of Zebu and crosses for draft power in the Kénédougou region in Burkina Faso. The time range and continuous usage of pure Zebu and Baule animals in the crossbred population are responsible for the a large spread of admixture levels in crosses. A region on CHR 22 (51.20–51.40 Mb) showed greatest admixture deviation in favor of Baoule when compared to the overall genome admixture. CHR 22 has been previously identified to have regions responsible for trypanotolerance (Hanotte et al., 2003; Gautier et al., 2009). Gautier et al. (2009) observed that a region of CHR 22 (region between 43.79–53.04 Mb), overlapping with our region contained a high proportion of SNPs under balancing selection. This result might be related to the maintenance of several haplotypes containing variants under positive selection within different populations. Alternatively, the fixation of the same variant in some populations could also lead to such a trend due to the low level of linkage disequilibrium across populations (Gautier et al., 2007, 2009). Genes involved in trypanotolerance on CHR 22 (between 51.20 and 51.40 Mb) such as MON1A, MST1R, UBA7, FAM212, CAMKV, TRAIP, CDHR4, IP6K1, RNF123, APEH, and MST1 (Supplementary Table 1) were described by O'Gorman et al. (2009). These authors found that trypanotolerant N'Dama cattle displayed a rapid and distinct transcriptional response to infection, with a 10-fold higher number of genes differentially expressed at day 14 post-infection compared to susceptible Boran cattle. Their analyses identified coordinated temporal gene expression changes for both breeds in response to trypanosome infection. Three other protein coding regions found on this part of CHR 22 (ENSBTAG00000040083, CDHR4 and AMIGO) did not show differential expression in their analysis.

One region, CHR 18 (17.10–17.30 Mb) showed significantly lower Baoule admixture than average admixture measured by microsatellites and SNPs. In a mapping study with experimental crosses of trypanotolerant N'Dama and trypanosusceptible Boran cattle, Hanotte et al. (2003) had found that in some instances the trypanosusceptible breed carried trypanotolerant alleles. This region contained one coding gene not previously described in trypanotolerance studies. Noyes et al. (2010) described a QTL on CHR 16 as region responsible in tolerant breeds to cope with anemia. On the region of CHR 16, MIA3, BROX, and AUH genes were found. Three protein coding sequences are present in the candidate region of CHR 21 (20.40–20.60 Mb): RLBP1, FANCI, and POLG, with RLBP1 and POLG showing differential expression. All four genes in the candidate region of chromosome 26 (HPS6, LDB1, NOLC1, and GBF1) were differentially expressed in the study of O'Gorman et al. (2009).

## Conclusions

In this study admixture in genomic regions potentially related to trypanotolerance was compared with admixture in the background genome. A non-significant trend of higher proportions of Baoule admixture in the candidate regions was found and a majority of regions (5 of 6) with admixture levels significantly different from background admixture indicated high levels of Baoule ancestry.

In this study, the discovery of trypanotolerance candidate regions was performed via a selection signature approach based on differences of allelic frequencies of trypanotolerant African taurine breeds versus other breeds around the world, using data from the Bovine HapMap consortium. Targeted SNP genotyping in candidate regions was the method of choice. Given the reduction of cost for high density SNP chip genotyping, part of the samples used in this study are now being genotyped with the commerical chip of Illumina Inc.,covering almost 800,000 SNPs. A large number of markers will also allow estimation of individual age of admixture and therefore the number of generations of natural selection acting on the composites. Targeted re-sequencing approaches of interesting candidate regions that can identify both common and exceedingly rare causal variants could potentially give more insight into trypanotolerance mechanisms.

Silbermayr et al. (2013) developed a novel qPCR assay for indication of infection status of animals with the three trypanosome species involved in AAT (*T. vivax, T. congolense*, and *T. brucei*) from blood samples of most of the animals involved in this study. Zebus were twice as often infected (21.74%) compared to Baoule (9.70%) and composites (9.57%). Phenotypic measures oftrypanosomosis by routine checking of infection status will help to identify best composites (Soudré et al., 2013). Information about best levels of admixture in composites is a premise of more effective and sustainable use of trypanotolerant types of cattle.

## Author contributions

JS conceived the study, with the support of OH. ASo collected samples and background information, as suggested by MW, GB, and MM provided genotyping facilities and support. The study performed to find trypanosoma tolerance region was performed by PB and ASt. Genotyping of bovine microsatellites was performed by ASo and SM, SNPs were genotyped by JB and ASo while KS genotyped parasites. Admixture and *F_ST_* analysis was performed by ASm who also drafted the manuscript. Results were interpreted by all authors, PB, JS, GM, and ASm provided the biggest contributions in manuscript revision. All authors read and approved the final manuscript. ASm – Anamarija Smetko; ASo – Albert Soudre; ASt – Alessandra Stella. The views expressed in this publication are those of PB and do not necessarily reflect the views or policies of FAO.

### Conflict of interest statement

The authors declare that the research was conducted in the absence of any commercial or financial relationships that could be construed as a potential conflict of interest.

## Supplementary material

Animal_breed_location_GPS_tryps.xlsx: Animal ID, Animal breed, Region, Village, Latitude, Longitude, Trypanosoma species present in blood sample of this animal.

SNP.ped: SNP data of 214 animals included in the study, 0 represents missing allele. First column is family ID column, followed by animal ID, sire ID, dam ID, sex and phenotype. Phenotype is denoted with -9 as missing and after this columns alleles for SNPs follow.

SNP.map: column with chromosome number, SNP name, Morgan position and bp positions of SNPs included in SNP.ped file.

MIC.ped: Row names are microsatellite names, first column is animal ID, followed by population code, flag code and location. After those columns microsatellites follow.

FAO-marker.xlsx: First column is name of microsatellite, second is chromosome, Genetic Map (MARC) and Sequence Map (STS).
