# Supplementary material for: Trypanosomosis: potential driver of selection in African cattle
Source: Front Genet. 2015 Apr 21;6:137. doi: 10.3389/fgene.2015.00137 (PMC4404968; doi:10.3389/fgene.2015.00137)
Supplement: Supplementary file 1 [file Presentation1.ZIP › Supplementary Data/Supplementary table 1.docx]

Supplementary Table 1. Coding regions found on candidate regions studied

| CHROMOSOME and position |  |  |  |
| --- | --- | --- | --- |
| (positions are based on *Btau 3.1 and |  |  |  |
| Btau 4.0) | Gene | Gene ID | Position of gene |
|  |  |  |  |
| 16 |  |  |  |
| *26851364-27107809 |  |  |  |
| ^$^2312967-23425355 | MIA3 | ENSBTAG00000018824 | *26803253-26862483 |
|  | AIDA | ENSBTAG00000007593 | *26862374-26908257 |
|  | BROX | ENSBTAG00000007595 | *26908537-26927548 |
|  | AUH | ENSBTAG00000044081 | *27058047-27058913 |
| 17 |  |  |  |
| *9921157-10140780 |  |  |  |
| ^$^10587595-10805467 |  |  |  |
|  |  | ENSBTAG00000027182 | *9742222-10173282 |
| 18 |  |  |  |
| *18195295-18344555 |  |  |  |
| ^$^17149842-17299102 |  |  |  |
|  | ZNF423 | ENSBTAG00000017397 | *18041277-18392207 |
| 20 |  |  |  |
| *18596290-18631431 |  |  |  |
| ^$^20005762-20040903 |  |  |  |
|  | DEPDC1B | ENSBTAG00000017026 | *18563739-18654726 |
| 20 |  |  |  |
| *21085469-21242156 |  |  |  |
| ^$^21934672-22159154 |  |  |  |
|  |  | ENSBTAG00000005229 | *20575856-20576238 |
|  | 5s-rRNA | ENSBTAG00000043799 | *20653612-20653737 |
| 21 |  |  |  |
| *21085469-21242156 |  |  |  |
| ^$^20437620-20594307 |  |  |  |
|  | bta-mir-2363-1 ENSBTAG00000044709 | | *21157504-21157577 |
|  | RLBP1 | ENSBTAG00000033721 | *21117499-21280811 |
|  | FANCI | ENSBTAG00000009097 | *21137918-21198618 |
|  | POLG | ENSBTAG00000009098 | *21197851-21215597 |
| 22 |  |  |  |

*20074083-20397580 ^$^20552362-2087872 22 *50887920-51087450 ^$^51223225-51422755

| CAMKV | ENSBTAG00000004878 | *50957515-50969031 |
| --- | --- | --- |
| TRAIP | ENSBTAG00000004877 | *50970363-50987947 |
| CDHR4 | ENSBTAG00000005728 | *51005176-51012628 |
| IP6K1 | ENSBTAG00000011595 | *51014686-51049408 |
| RNF123 | ENSBTAG00000011588 | *51054914-51078183 |
| AMIGO3 | ENSBTAG00000045547 | *51056714-51058249 |
| APEH | ENSBTAG00000011583 | *51086070-51094554 |
| MST1 | ENSBTAG00000011585 | *51080758-51085659 |

26

*22610550-22826271 ^$^23191018-23406739

| HPS6 | ENSBTAG00000021942 | *22623887-22626506 |
| --- | --- | --- |
| LDB1 | ENSBTAG00000005780 | *22652090-22663974 |
| PPRC1 | ENSBTAG00000007427 | *22677211-22691109 |
| NOLC1 | ENSBTAG00000007435 | *22692692-22701499 |
| GBF1 | ENSBTAG00000006014 | *22761606-22884208 |
| ELOVL3 | ENSBTAG00000015700 | *22742695-22745622 |
| PITX3 | ENSBTAG00000015702 | *22745904-22757812 |
| SNORD22 | ENSBTAG00000042186 | *22676069-22676182 |
|  |  |  |
